# Prognostic value of the ratio of maximum to minimum diameter of primary tumor in metastatic clear cell renal cell carcinoma

**DOI:** 10.1186/s12894-022-01047-y

**Published:** 2022-07-04

**Authors:** Hongzhe Shi, Chuanzhen Cao, Li Wen, Lianyu Zhang, Jin Zhang, Jianhui Ma, Jianzhong Shou, Changling Li

**Affiliations:** 1 Department of Urology, National Cancer Center/National Clinical Research Center for Cancer/Cancer Hospital, Chinese Academy of Medical Sciences and Peking Union Medical College, Panjiayuan Nanli 17#, Chaoyang District, Beijing, 100021 People’s Republic of China; 2 Department of Imaging, National Cancer Center/National Clinical Research Center for Cancer/Cancer Hospital, Chinese Academy of Medical Sciences and Peking Union Medical College, Beijing, People’s Republic of China

**Keywords:** Tumor morphology, Prognosis, Ratio of maximum to minimum tumor diameter, Metastatic renal cell carcinoma

## Abstract

**Background:**

Several models and markers were developed and found to predict outcome of advanced renal cell carcinoma. This study aimed to evaluate the prognostic value of the ratio of maximum to minimum tumor diameter (ROD) in metastatic clear cell renal cell carcinoma (mccRCC).

**Methods:**

Patients with mccRCC (n = 213) treated with sunitinib from January 2008 to December 2018 were identified. Cutoff value for ROD was determined using receiver operating characteristic. Patients with different ROD scores were grouped and evaluated. Survival outcomes were estimated by Kaplan–Meier method.

**Results:**

The optimal ROD cutoff value of 1.34 was determined for progression free survival (PFS) and overall survival (OS). Patients in ROD ≥ 1.34 group had shorter PFS (9.6 versus 17.7 months, p < 0.001) and OS (25.5 versus 32.6 months, p < 0.001) than patients in ROD < 1.34 group. After adjustment for other factors, multivariate analysis showed ROD ≥ 1.34 was an independent prognostic factor for PFS (p < 0.001) and OS (p = 0.006). Patients in ROD ≥ 1.34 group presented higher proportions of pT3/4 stage (89.2% versus 10.8%, p = 0.021), WHO/ISUP grade III/IV (72.0% versus 28.0%, p = 0.010), tumor necrosis (71.0% versus 29.0%, p = 0.039), sarcomatoid differentiation (79.1% versus 20.9%, p = 0.007), poor MSKCC risk score (78.4% versus 21.6%, p < 0.001) and poor IMDC risk score (74.4% versus 25.6%, p < 0.001) than ROD < 1.34 group.

**Conclusion:**

Primary tumor with higher ROD was an independently prognostic factor for both PFS and OS in patients with mccRCC who received targeted therapy. Higher ROD was also associated with high pT stage, high WHO/ISUP grade, sarcomatoid features, tumor necrosis, poor MSKCC and IMDC risk score.

**Supplementary Information:**

The online version contains supplementary material available at 10.1186/s12894-022-01047-y.

## Background

Renal cell carcinoma (RCC) accounts for approximately 3% of malignant tumors in adults and 20% to 30% of them are diagnosed as advanced diseases with poor prognosis [1, 2]. Targeted therapy has been the standard metastatic RCC treatment since 2005 [3, 4]. Several risk score models, such as the Memorial Sloan Kettering Cancer Center (MSKCC) system and the International Metastatic Renal Cell Carcinoma Database Consortium (IMDC) criteria, have been found and validated to predict the prognosis of different patients with metastatic RCC [5, 6]. However, MSKCC and IMDC classification mostly focus on the state of the performance status and laboratory values while the primary tumor status has not been involved in.

Several studies have reported primary tumor size may be associated with prognosis of metastatic RCC [7, 8]. It is still controversial and cannot completely reflect the role of primary tumor. The ratio of maximum to minimum tumor diameter (ROD), a specific primary tumor feature, has not been mentioned. Previously, we have investigated the utility of ROD in predicting pathologic subtypes of RCC before surgery and found its association with adverse pathological factors [9]. We used ROD to quantify tumor irregularity. The more irregular the tumor, the higher the degree of malignancy was discovered. To further demonstrate the prognostic value of the ROD, in this study, we retrospectively analyzed the records of clear cell RCC (ccRCC) patients treated by surgery and followed sunitinib due to subsequent or simultaneous metastasis.

## Patients and methods

### Patient selection

This is a retrospective study focusing on patients with metastatic ccRCC (mccRCC), which was approved by the Cancer Hospital Chinese Academy of Medical Sciences of Ethics Committee (ID: NCC2016YJC-08). Patient consent for treatment and follow-up was included in each medical record. Patients diagnosed as ccRCC after surgery and treated with sunitinib for metastasis between January 2008 and December 2018 were collected. Metastasis was confirmed by imaging examination. Sunitinib was initially administered 50 mg once daily, on a 4/2 schedule. Patients received other systematic therapies after sunitinib treatment failure, including pazopanib, everolimus, axitinib, immune checkpoint inhibitors, or other free second-line therapy trials. Clinicopathological features, such as age, gender, Karnofsky performance status, presenting symptom, tumor size, World Health Organization/International Society of Urologic Pathologists (WHO/ISUP) grade, tumor necrosis, sarcomatoid differentiation, MSKCC and IMDC criteria were used to evaluate.

### Radiological assessment

Patients had contrast-enhanced computed tomography (CT) or magnetic resonance imaging (MRI) examination within 2 weeks before surgery for primary tumor diagnosis and clinical staging. CT scans were undertaken every 4–8 week since taking sunitinib, and the Response Evaluation Criteria in Solid Tumors (RECIST) criteria version 1.1 were used to evaluate [10]. The best response of treatment, including complete response (CR), partial response (PR), stable disease (SD) and progression disease (PD) was recorded.

**Fig. 1:**
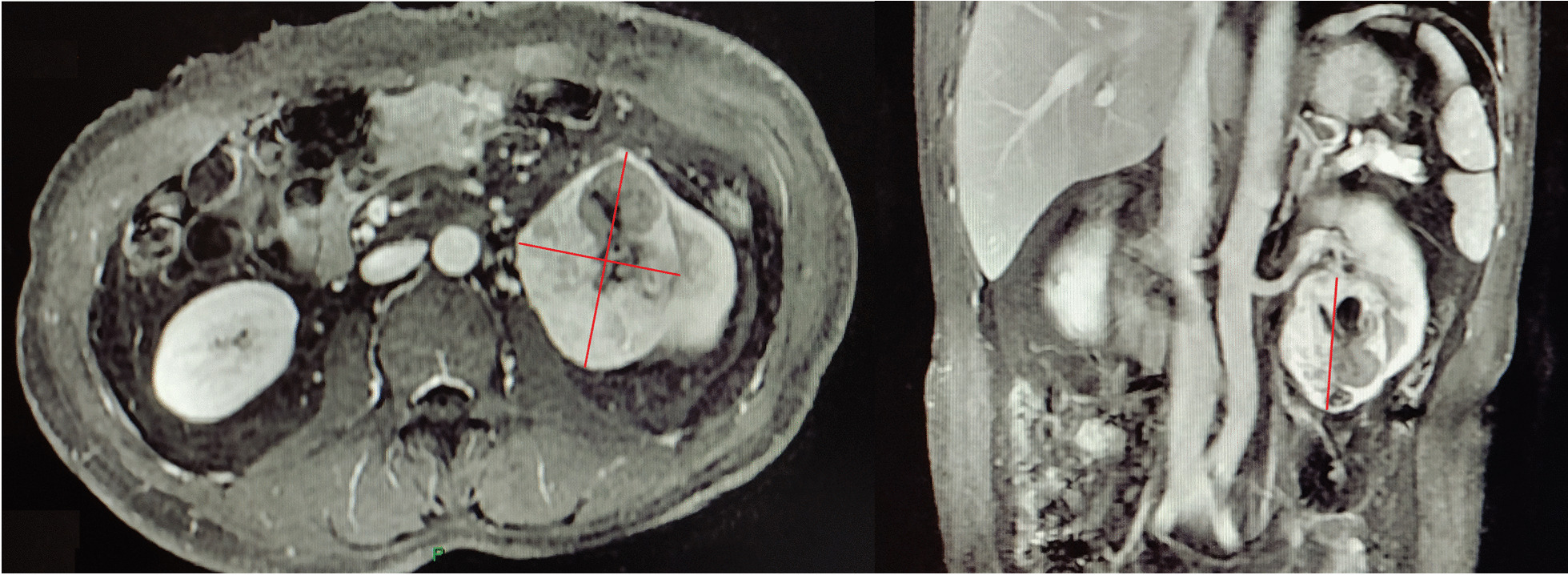
Example of measuring the three diameters: one maximum, one sub maximum, and one minimum

The diameters of the tumor were measured including coronal, sagittal and axial view, including a maximum diameter, a sub maximum diameter and a minimum diameter in clinical staging before surgery (Fig. 1). Tumor’s three diameters were recorded separately by 2 independent radiologists. The ratio of the maximum diameter to the minimum diameter (ROD) was calculated to quantify tumor irregularity.

### Statistical analysis

Chi-square test, Fisher’s exact test or Student’s t-test were used to analyze the relationship of different groups. The optimal cutoff value of ROD in prediction of survival outcomes was determined by Receiver Operating Characteristic (ROC) curve analysis. To investigate the survival outcome of patients with mccRCC, progression-free survival (PFS) and overall survival (OS) from the initiation of sunitinib were determined using the Kaplan–Meier method and were analyzed using the log-rank test. Cox proportional hazards regression models were used to assess the significant factors unadjusted and adjusted for covariates. Statistical analysis was performed using SPSS 23.0 software, and differences were considered statistically significant if p values were < 0.05.

## Results

### Patients’ characteristics

A total of 213 patients with a median age of 55.0 years (range, 17–76 years) were identified. Patients included 139 (65.3%) males and 74 (34.7%) females. Median primary tumor size measured in CT or MRI was 6.1 cm (range, 1.1–15.6 cm). The number of patients in cT1/2, cT3/4 stage was 199 (93.4%), 14 (6.6%), respectively. All patients were received nephrectomy before systemic therapy. Patients consisted of 96 (45.1%) cases of synchronous RCC and 117 (54.9%) cases of metachronous RCC. The number of patients in pT1/2, pT3/4 stage was 188 (88.3%), 25 (11.7%), respectively. The most common site of metastasis was pulmonary in 164 cases (77.0%), followed by lymph node metastasis in 86 cases (40.4%). Other metastases included bone, adrenal, liver, brain, spleen, and pancreas. There were 152 patients (71.4%) harboring more than 2 metastatic organs. Twenty-two patients (10.3%) had responders including CR (n = 5, 2.3%) and PR (n = 17, 8.0%) during the treatment of sunitinib. Patients’ characteristics were summarized in Table 1.

**Table 1:** Correlation between ROD and the clinicopathological features of patients with metastatic renal cell carcinoma

Characteristics	Total	ROD < 1.34 (n = 83)	ROD ≥ 1.34 (n = 130)	p value
Age at sunitinib (mean ± SD)	53.3 ± 9.8	52.1 ± 10.7	54.1 ± 9.2	0.139
Gender, n (%)				0.961
Man	139 (65.3)	54 (38.8)	85 (61.2)	
Woman	74 (34.7)	29 (39.2)	45 (60.8)	
KPS score < 80, n (%)	15 (7.0)	5 (33.3)	10 (66.7)	0.643
Presenting symptom, n (%)	19 (8.9)	7 (36.8)	12 (63.2)	0.842
Tumor size (mean ± SD)	6.3 ± 2.2	6.7 ± 2.1	6.1 ± 2.2	0.089
Tumor location, n (%)				0.182
Left	102 (47.9)	35 (34.3)	67 (65.7)	
Right	111 (52.1)	48 (43.2)	63 (56.8)	
Type of metastasis				0.908
Synchronous	96 (45.1)	37 (24.0)	59 (76.0)	
Metachronous	117 (54.9)	46 (51.3)	71 (48.7)	
cT stage, n (%)				0.012
cT1/2	199 (93.4)	82 (41.2)	117 (58.8)	
cT3/4	14 (6.6)	1 (7.1)	13 (92.9)	
pT stage, n (%)				0.021
pT1/2	188 (88.3)	82 (38.5)	106 (49.8)	
pT3/4	25 (11.7)	1 (0.5)	24 (11.2)	
WHO/ISUP grade, n (%)				0.010
I/II	131 (61.5)	60 (45.8)	71 (54.2)	
III/IV	82 (38.5)	23 (28.0)	59 (72.0)	
Tumor necrosis, n (%)	69 (32.4)	20 (29.0)	49 (71.0)	0.039
Sarcomatoid differentiation, n (%)	43 (20.2)	9 (20.9)	34 (79.1)	0.007
MSKCC risk classification				< 0.001
Good	102 (47.9)	59 (57.8)	43 (42.2)	
Intermediate and poor	111 (52.1)	24 (21.6)	87 (78.4)	
IMDC risk classification				< 0.001
Good	92 (43.2)	52 (56.5)	40 (43.5)	
Intermediate and poor	121 (56.8)	31 (25.6)	90 (74.4)	
Number of metastatic organs				0.943
< 2	61 (28.6)	24 (39.3)	37 (60.7)	
≥ 2	152 (71.4)	59 (38.8)	93 (61.2)	
Response to sunitinib, n (%)				0.844
Responder	22 (10.3)	9 (40.9)	13 (59.1)	
Non-responder	191 (89.7)	74 (38.7)	117 (61.3)	

*ROD* Ratio of maximum to minimum tumor diameter *KPS* Karnofsky performance status, *WHO/ISUP* World Health Organization/International Society of Urologic Pathologists, *MSKCC* Memorial Sloan Kettering Cancer Center, *IMDC* International Metastatic Renal Cell Carcinoma Database Consortium

### The association between ROD and clinicopathological features

ROC curves were constructed to determine the appropriate cutoff point of ROD (Additional file 1: fig S1). The most discriminative ROD cutoff value of 1.34 was selected for both PFS (sensitivity = 79.3%, specificity = 74.0%; Area Under Curve, AUC = 0.810, 95% confidence interval, CI:0.752–0.869, p < 0.001) and OS (sensitivity = 84.9%, specificity = 83.8%; AUC = 0.932, 95% CI:0.899–0.966, p < 0.001). The patients were divided into two groups including ROD < 1.34 group (n = 83) and ROD ≥ 1.34 group (n = 130). Comparison between ROD and the Clinicopathological features were showed in Table 1. Patients in ROD ≥ 1.34 group presented higher proportions of pT3/4 stage (89.2% versus 10.8%, p = 0.021), WHO/ISUP grade III/IV (72.0% versus 28.0%, p = 0.010), tumor necrosis (71.0% versus 29.0%, p = 0.039), sarcomatoid differentiation (79.1% versus 20.9%, p = 0.007), poor MSKCC risk score (78.4% versus 21.6%, p < 0.001) and poor IMDC risk score (74.4% versus 25.6%, p < 0.001) than ROD < 1.34 group.

### The evaluation of ROD in clinical outcomes

**Fig. 2:**
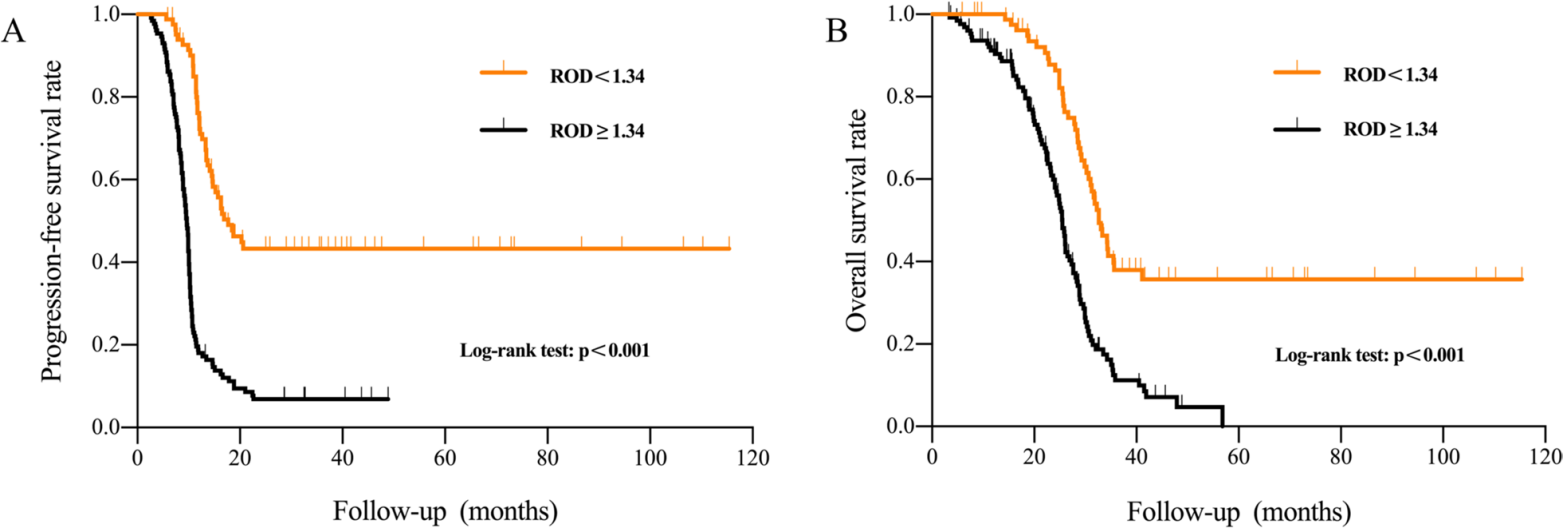
Patients with ROD ≥ 1.34 had shorter progression free survival (**A**) and overall survival (**B**) than patients with ROD < 1.34

After 32.0 months (range, 2.6–125.8 months) median follow-up, patients in ROD ≥ 1.34 group experienced significantly shorter PFS (median, 9.6 versus 17.7 months, p < 0.001) and OS (median, 25.5 versus 32.6 months, p < 0.001) than patients in ROD < 1.34 group (Fig. 2).

The univariate survival analysis revealed that ROD ≥ 1.34 (p < 0.001), WHO/ISUP grade (p < 0.001), tumor necrosis (p = 0.032), sarcomatoid differentiation (p < 0.001), MSKCC score (p < 0.001) and IMDC score (p < 0.001) appeared as significant prognostic factors for PFS (Table 2). After adjustment for WHO/ISUP grade (p < 0.001), tumor necrosis (p = 0.118), sarcomatoid differentiation (p = 0.035), MSKCC score (p = 0.008) and IMDC score (p < 0.001), the multivariate Cox regression analysis revealed that ROD ≥ 1.34 (adjusted hazard ratio, HR = 3.068, 95% CI:2.102–4.478, p < 0.001) was an independent prognostic factor for poor PFS (Table 3). In addition, presenting symptom (p = 0.007), ROD ≥ 1.34 (p < 0.001), WHO/ISUP grade (p < 0.001), tumor necrosis (p = 0.007), sarcomatoid differentiation (p = 0.016), MSKCC score (p < 0.001) and IMDC score (p < 0.001) were prognostic factors for OS (Table 2). After adjustment for presenting symptom (p = 0.009), WHO/ISUP grade (p < 0.001), tumor necrosis (p < 0.001), sarcomatoid differentiation (p = 0.621), MSKCC score (p = 0.002) and IMDC score (p < 0.001), ROD ≥ 1.34 (adjusted HR = 1.774, 95% CI:1.175–2.676, p = 0.006) was still an independent prognostic factors for OS (Table 3).

**Table 2:** Univariate Cox regression analysis of clinical factors in patients with metastatic renal cell carcinoma

Variable	Progression free survival			Overall survival		
	HR	95% CI	p value	HR	95% CI	p value
Age	1.003	0.988–1.019	0.653	1.001	0.985–1.018	0.871
Gender, male	0.936	0.677–1.293	0.688	0.829	0.586–1.173	0.290
KPS, < 80	1.064	0.520–2.177	0.864	0.577	0.212–1.569	0.281
Presenting symptom	1.292	0.770–2.168	0.332	2.256	1.247–4.082	0.007
Tumor location, left	1.027	0.753–1.400	0.867	1.073	0.769–1.497	0.678
ROD, ≥ 1.34	3.974	2.784–5.673	< 0.001	2.783	1.927–4.018	< 0.001
Number of metastatic organs, ≥ 2	1.053	0.728–1.547	0.468	0.459	0.592–1.426	0.584
pT stage, 3/4	1.021	0.677–1.676	0.061	1.025	0.823–1.312	0.078
WHO/ISUP grade, III/IV	2.308	1.684–3.165	< 0.001	3.164	2.249–4.415	< 0.001
Tumor necrosis	1.438	1.031–2.007	0.032	1.677	1.167–2.410	0.007
Sarcomatoid differentiation	2.131	1.455–3.120	< 0.001	1.722	1.107–2.680	0.016
MSKCC score, intermediate and poor	2.624	1.895–3.634	< 0.001	3.125	2.174–4.493	< 0.001
IMDC score, intermediate and poor	3.342	2.116–6.528	< 0.001	4.317	2.383–6.657	< 0.001

*KPS* Karnofsky performance status *ROD* Ratio of maximum to minimum tumor diameter, *WHO/ISUP* World Health Organization/International Society of Urologic Pathologists, *MSKCC* Memorial Sloan Kettering Cancer Center, *IMDC* International Metastatic Renal Cell Carcinoma Database Consortium

**Table 3:** Independent prognostic factors analyzed using a multivariable Cox model in patients with metastatic renal cell carcinoma

Variable	Progression free survival			Overall survival		
	HR	95% CI	p value	HR	95% CI	p value
Presenting symptom				2.219	1.220–4.037	0.009
ROD, ≥ 1.34	3.068	2.102–4.478	< 0.001	1.774	1.175–2.676	0.006
WHO/ISUP grade, III/IV	2.102	1.512–2.922	< 0.001	2.699	1.880–3.874	< 0.001
Tumor necrosis	1.315	0.933–1.854	0.118	2.046	1.395–3.002	< 0.001
Sarcomatoid differentiation	1.529	1.031–2.268	0.035	1.123	0.710–1.776	0.621
MSKCC score, intermediate and poor	1.619	1.137–2.305	0.008	1.901	1.261–2.864	0.002
IMDC score, intermediate and poor	2.315	1.427–4.438	< 0.001	2.004	1.258–4.062	< 0.001

*ROD* Ratio of maximum to minimum tumor diameter, *WHO/ISUP* World Health Organization/International Society of Urologic Pathologists, *MSKCC* Memorial Sloan Kettering Cancer Center, *IMDC* International Metastatic Renal Cell Carcinoma Database Consortium

## Discussion

From published papers, intratumor heterogeneity is a key factor contributing to the survival of cancer, therapeutic failure, and drug resistance 11, 12]. The scale of heterogeneity within a tumor has also been found and proved in RCC [13, 14]. Studies have showed that intratumor heterogeneity may contribute to the polyclonal growth pattern of tumors [15]. In addition, polyclonal growth commonly accompanies with corresponding change of tumor microenvironment, which is also thought to play an important role for tumor growth and progression and to be involved in the treatment outcome of targeted therapy [16]. We considered that the intratumor heterogeneity evolved to the asymmetric tumor morphology. From the clinical practice, we observed that the ROD could be used to quantify the tumor irregularity. The higher the intratumor heterogeneity, the more irregular tumor and the higher ROD would achieve, which was likely to reveal the efficacy of later treatment.

The ROD, a specific feature of primary tumor, has not been investigated before. In this study, the most optimal ROD cutoff value of 1.34 was determined. Patients in ROD ≥ 1.34 group accounted for 61.0% (130/213). And we were not surprised to find that more cases of metastatic renal cell carcinoma were in the group with larger ROD. This finding suggested a new hypothesis that the stronger tumor heterogeneity have more chances of metastasis. In this study, patients of pT1/2 accounted for 88.3% which was more than pT3/4. Patients of pT1/2 increased because of the popularity of health examination. Although patients of pT3/4 were more easily to occur metastasis, pT1/2 had large base population which resulted in pT1/2 had a high proportion in metastatic patients. Another reason was that all patients enrolled were able to receive nephrectomy and unresectable T3/4 patients were excluded.

We found that the ROD ≥ 1.34 was also significantly associated with prognosis. Compared with patients in ROD ≥ 1.34 group, patients in ROD < 1.34 group achieved longer PFS (17.7 versus 9.6 months, p < 0.001) and OS (32.6 versus 25.5 months, p < 0.001). Variable pathology factors including WHO/ISUP grade, tumor necrosis, and sarcomatoid differentiation were also associated with outcome of RCC, which were confirmed in previous studies [17–19]. In this study, these features were also investigated. WHO/ISUP grade, sarcomatoid differentiation, MSKCC score and IMDC score were independent prognostic factors for PFS, and presenting symptom, WHO/ISUP grade, tumor necrosis, MSKCC score and IMDC score were independent prognostic factors for OS. Interestingly, we found that patients in ROD ≥ 1.34 group were more likely to present high WHO/ISUP grade, high pT stage, tumor necrosis, sarcomatoid differentiation, poor MSKCC risk score, and poor IMDC score. This showed that as an index to quantify tumor irregularity, ROD had a strong relationship with tumor malignancy. Besides above findings, we also found that ROD ≥ 1.34 was an independent prognostic factor for both poor PFS and OS.

Our study includes several limitations that need to be acknowledged. It was a retrospective study including limited cases, which still needed to accumulate data, and we will carry out prospective studies to confirm. In addition, we will further explore the possible molecular mechanism of poor outcome associated with irregularity of primary tumor.

## Conclusions

Primary tumor with ROD ≥ 1.34 was an independently prognostic factor for both PFS and OS in patients with mccRCC who received targeted therapy and also associated with high pT stage, high WHO/ISUP grade, sarcomatoid features, tumor necrosis, poor MSKCC and IMDC risk score. Further prospective validation is required to confirm these findings.

## Supplementary Information

**Additional file 1:** **Fig. S1.** Receiver operating characteristics curves were used to determine the appropriate cut-off point of ROD predicting survival. (A) progession free survival and (B) overall survival.

## Data Availability

The datasets generated and/or analyzed during the current study are not publicly available due to individual privacy could be compromised but are available from the corresponding author on reasonable request.

## Abbreviations

AUC: Area under curve
CI: Confidence interval
CR: Complete response
CT: Computed tomography
HR: Hazard ratio
IMDC: International metastatic renal cell carcinoma database consortium
mccRCC: Metastatic clear cell renal cell carcinoma
mRCC: Metastatic RCC
MRI: Magnetic resonance imaging
MSKCC: Memorial sloan kettering cancer center
NCC/CHCAMS: National cancer center/cancer hospital, chinese academy of medical sciences
OS: Overall survival
PD: Progression disease
PFS: Progression-free survival
PR: Partial response
RCC: Renal cell carcinoma
RECIST: Response evaluation criteria in solid tumors
ROC: Receiver operating characteristic
ROD: Ratio of maximum to minimum tumor diameter
SD: Stable disease
WHO/ISUP: World health organization/international society of urologic pathologists
