# Supplementary figures and images for: Prognostic value of the ratio of maximum to minimum diameter of primary tumor in metastatic clear cell renal cell carcinoma
Source: BMC Urol. 2022 Jul 4;22:95. doi: 10.1186/s12894-022-01047-y (PMC9252060; doi:10.1186/s12894-022-01047-y)

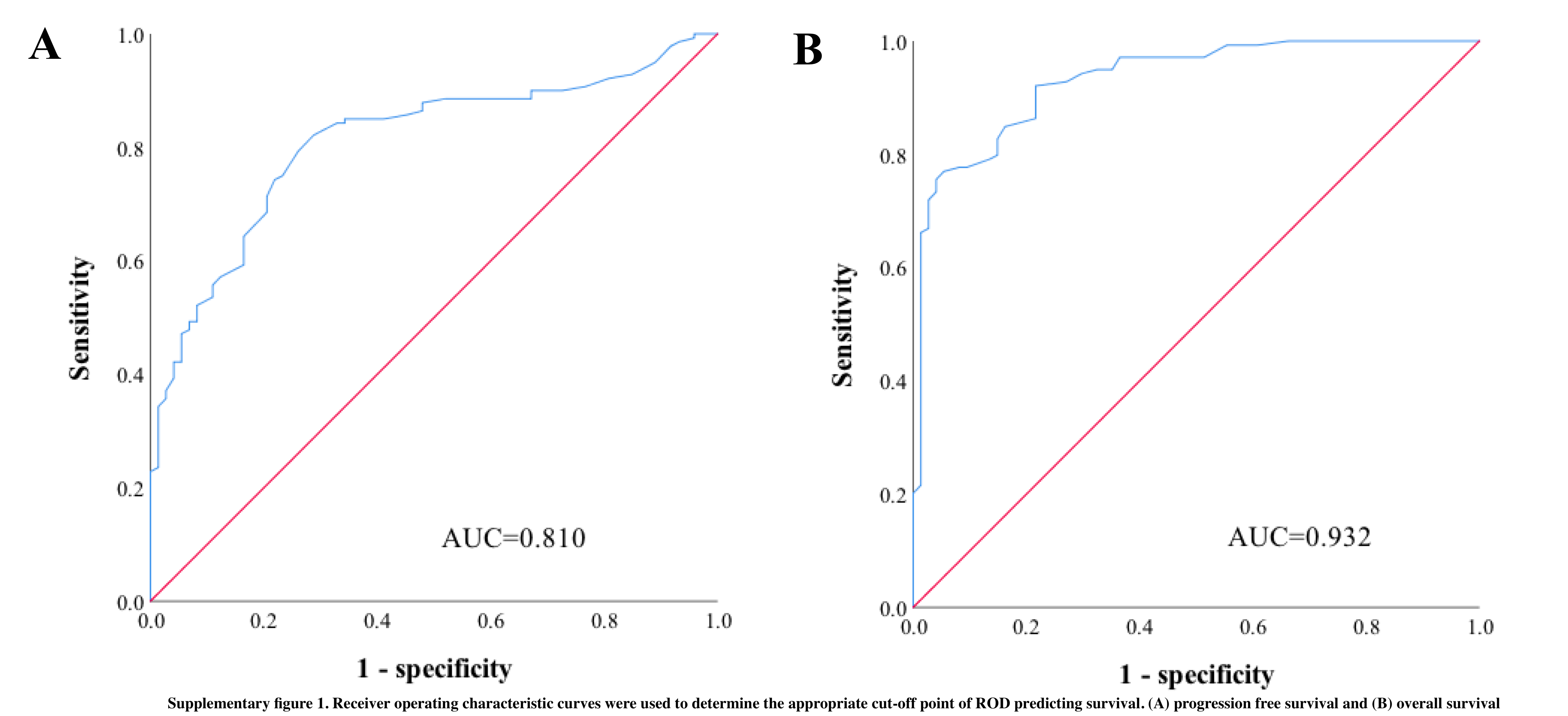

Supplement: Supplementary file 1 — Additional file 1: Fig. S1. Receiver operating characteristics curves were used to determine the appropriate cut-off point of ROD predicting survival. (A) progession free survival and (B) overall survival. [file 12894_2022_1047_MOESM1_ESM.tif]
